# Dentate Gyrus activation during visual but not auditory pattern separation: an investigation of perceptual modality differences in young and old individuals using fMRI

**DOI:** 10.3389/fpsyt.2026.1900423

**Published:** 2026-09-24

**Authors:** Marko Rajkovic, Else Schneider, Marco Phil Caviezel, Rudolf Krug, Carolin Franziska Reichert, Stefan Borgwardt, Christoph Linnemann, Annette Beatrix Brühl, Undine Lang, Tobias Melcher

**Affiliations:** 1 Research Cluster Molecular and Cognitive Neurosciences, Department of Biomedicine University of Basel, Basel, Switzerland; 2 Division of Cognitive Neuroscience, Department of Biomedicine, University of Basel, Basel, Switzerland; 3 Psychiatric University Hospital (UPK), University of Basel, Basel, Switzerland; 4 Centre for Chronobiology, Psychiatric University Clinics (UPK), University of Basel, Basel, Switzerland; 5 Translational Psychiatry Unit (TPU), Department of Psychiatry and Psychotherapy, University of Lübeck, Lübeck, Germany

**Keywords:** fMRI, healthy aging, mnemonic similarity task, pattern separation, visual and auditory perception

## Abstract

**Introduction:**

Pattern separation (PS) is a neurocognitive process that is essential for distinguishing highly similar objects from one another. The dentate gyrus has been previously considered the primary anatomical and neurofunctional focus of this central memory process. PS has traditionally been studied in the context of visual discrimination of highly similar everyday objects. To date, the neurofunctional substrates of auditory PS have not been investigated, and only a handful of studies have examined the behavioural age effects on auditory PS.

**Methods:**

The present study adapted the prominent mnemonic similarity task from Stark et al. (2019) to assess and directly compare both visual and auditory PS using fMRI. A sample of 46 subjects, 23 per group, aged between 19 and 79 years of age, completed alternating trials of visual and auditory pattern separation using non-semantic visual shapes and two-syllabic pseudowords.

**Results:**

Contrary to our expectations, no hippocampal activation was found during auditory PS. Instead, left-lateralized cortical regions were identified, including the superior temporal gyrus, the inferior frontal gyrus, and the pre-supplementary motor area. Furthermore, the older subjects demonstrated age-related deficits in lure discrimination and recognition memory performance; however, these deficits were not consistently observed at the neurofunctional level.

**Discussion:**

In sum, the present findings point to a double dissociation between visual and auditory PS. Furthermore, the results on ageing, both behavioural and functional, could reflect age-related changes in neurovascular coupling and declines in visual and auditory perception, as well as methodological challenges in examining PS across different sensory modalities and age.

## 1 Introduction

Pattern separation (PS) is a prominent neural mechanism that involves discriminating between highly similar sensory representations. In its function, PS reduces overriding and interference during information encoding and is considered an important step prior to consolidation into long-term memory (1). Generally, during information processing, novel sensory input is continuously related to information stored in long-term memory. Thereby, the degree of sensory similarity or dissimilarity between the corresponding representations can either initiate PS, or a process orthogonal to PS referred to as pattern completion (PC) (2). The main objective of PC is to allow accurate generalisation in the presence of noise or partial sensory input (2). Taken together, PS and PC are complementary mechanisms in the cognitive processing of overlapping representations, yielding antagonistic outcomes: discrimination and equalisation, respectively.

Traditionally, PS functioning has been investigated using the mnemonic similarity task, sometimes referred to as the behavioural pattern separation (BPS) task (3). The task involves an encoding phase followed by a surprise recognition phase. During encoding, participants are usually shown a series of everyday objects. During recognition, the participants are shown a sequence of pictures: (1) one third consists of the same pictures shown during encoding, which are referred to as Targets. (2) One third consists of completely new images, previously not seen by the subjects, called Foils. (3) The last third consists of similar-looking but not identical images to those shown in the encoding task, named Lures. Since its inception, the mnemonic similarity task has spurred numerous studies that have consistently linked PS to the hippocampus.

Accordingly, neuroscientific investigations in recent years characterise PS as a fundamental hippocampal process and thereby highlight the contributions of specific hippocampal subfields, notably CA3 and, in particular, the dentate gyrus (DG), a subregion of the hippocampus that is located on the medial rim of the hippocampus (1, 4, 5). This role of the DG as a pattern separator has been replicated numerous times in animal lesion and stimulation studies (cf. 6), as well as in a substantial body of neuroimaging and biomarker work in human subjects (2, 7, 8). Furthermore, the DG has been linked to adult hippocampal neurogenesis, a process that produces new adult neurons even in advanced stages of aging, and has been shown to improve PS performance if stimulated, as demonstrated in animals (9, 10) However, despite its restorative capabilities, the DG and PS, in particular, are adversely affected by Ageing, marked by a profound structural decline in the DG of aged individuals. These structural reductions in DG volume have been associated with diminishing PS performance, observed in both humans (11, 12) and animals (12, 13). In this context, structural reductions in DG volume can lead to a functional imbalance within the hippocampal DG-CA3 network (14), causing a general shift from PS to PC with age (15). Interestingly, this decline in PS performance is contrasted by an overall intact object recognition performance in healthy aged adults (16). This suggests that PS is a highly specific marker for naturally occurring age-related changes in the brain. Beyond “healthy” lifespan development, other studies show an additional reduction in PS performance associated with neurodegeneration, spanning both pre-clinical and clinical stages of ageing (17, 18). In summary, PS appears to be a sensitive and valid cognitive marker for both pathological (i.e., Alzheimer-related) neurodegeneration and non-pathological alterations in the neural memory system across the human lifespan.

In general, previous evidence on PS has been primarily gathered in the context of the visual mnemonic similarity task or a variation thereof (cf. Stark et al. (3)). Accordingly, the cumulative literature offers limited insight into the neurocognitive functioning of PS in other sensory domains. However, a coherent series of animal studies has demonstrated that the DG plays an essential role not only in discriminating highly similar visual representations but also in discriminating olfactory representations, such as odours, in conjunction with the olfactory bulb and the piriform cortex. (6, 19). In humans, evidence suggests that increased hippocampal volume may facilitate odour discrimination performance (20). Furthermore, PS neural functioning remains under investigation in other sensory systems, such as the auditory system. To our knowledge, only a handful of imaging studies have examined auditory PS; however, they are exclusively EEG-based, focusing on mismatch negativity rather than fMRI (21, 22). In addition, only one study, to date, has investigated behavioural auditory pattern separation across age. However, the comparison was between auditory PS and statistical learning, rather than between visual PS and another sensory domain, and no brain imaging was conducted (23). Thus, it remains a subject for investigation whether visual and auditory PS rely on the same neural substrates when compared directly using fMRI.

Taken together, the previous evidence suggests that the hippocampus, particularly the DG, could serve as a neural substrate for a domain-independent, superordinate neurofunctional mechanism of PS across sensory modalities. In this sense, the present study had two primary aims. First, it aims to determine whether auditory PS recruits the same or different neural substrates as visual PS when operationalised in an analogous manner. Second, it aims to elucidate and compare naturally occurring age-related neurofunctional changes between both perceptual modalities in a population of healthy young and healthy old subjects. For the outlined study purposes, we adapted the prominent MST paradigm, which enables equivalent, and thus fully comparable, operationalisations of visual and auditory PS.2.

## Materials and methods

### 2.1 Participants

Our sample consisted of 48 healthy German-native speakers (29 females; 43 right-handed). To asses age-related changes in the neural substrates of both visual and auditory PS the sample was divided into a young (mean age: 23.39 years; age range: 19–31 years) and old group (mean age: 68.58 years; age-range: 59–79 years). All subjects reported no personal or first-degree relative history of psychiatric or neurological disorders, and had normal or corrected-to-normal visual acuity and sufficient hearing. In addition, all subjects belonging to the old group had been screened for signs of neurocognitive decline, including MCI and dementia using the Mini-Mental State Examination (MMSE) (**Supplementary Table 1**). All subjects provided written informed consent before participating and received either university course credits or a financial incentive for their time during the study. However, two subjects were excluded prior to analysis. One subject was excluded from further analysis due to an incidental finding, and another due to insufficient data. In total, the final sample for further analysis comprised 46 subjects. The complete project, including the experimental procedure, was approved by the local ethics committee (EKNZ – Ethikkommission Nordwest- und Zentralschweiz; http://www.swissethics.ch) and conducted in accordance with the declaration of Helsinki.

### 2.2 Study procedure

Participants were scanned at the University Hospital Basel (Clinic of Radiology and Nuclear Medicine) and were given a questionnaire in which they provided demographic information and details regarding MR safety procedures. All task instructions were given orally prior to the actual scanning. In addition to the verbal instructions, participants completed a practice trial outside of the scanner. For this purpose, a unique subset of trial stimuli was shown only during the training phase. In order to reduce mnemonic load, prior to the scanning, subjects were instructed to memorise six specific cue stimuli, three visual and three auditory stimuli, that would subsequently appear during the actual experiment.

### 2.3 Task

The current study adapted the prominent mnemonic-similarity task previously used by Stark et al. to assess PS in both behavioural and fMRI studies (3, 16). Both the experimental stimulation and the behavioural data acquisition were implemented in E-Prime (Version 2.0, Psychology Software Tools, Pittsburgh, PA, USA) using headphones and a two-button panel. Visual stimuli were presented on a projector screen located at the end of the MR scanner and viewed via a mirror system mounted on the head coil, while auditory stimuli were presented only acoustically via headphones. Each task block was initialised with a text display lasting 2800 ms that indicated whether the next block would be visual or auditory. After that, a cue stimulus was presented, followed by five to seven test stimuli (probes). To facilitate performance, a red frame indicated to the subjects that the stimulus presented was a cue, while a black frame indicated that it was a probe. Subjects then ought to indicate for each probe whether it was identical or different to the preceding cue using a two-button panel. All participants completed 216 trials, equally divided between visual and auditory PS tasks.

The different cue-probe combinations provided the same three main experimental conditions in both modalities, represented by 36 trials each: (1) the same condition, in which the probe was identical to the cue stimulus, (2) the lure condition, in which the probe was highly similar but not identical to the cue, and (3) the distinct condition in which the probe was not previously shown and clearly differed from the corresponding cue. Depending on the specific cue-probe combinations, the same stimulus could serve as both the lure and the distinct condition, which was counterbalanced across stimuli throughout the experiment. We recorded the button-press response to the probe throughout the entire presentation period. Simultaneously, we recorded each subject’s reaction time and response accuracy to each stimulus presentation. Each Cue stimulus was presented for 3000ms, while all probes were presented for 1800ms. The inter-stimulus interval consisted of the presentation of a fixation cross and was systematically jittered from 500ms to 900ms to improve event separation and the efficiency of the hemodynamic response estimation (for trial timing and composition, see **Figure 1**). The order of presentation was equally pseudorandomised for both visual and auditory blocks and counterbalanced for n-1 trial transitions, as demonstrated by the equal distribution of same, distinct, and lure trials following a lure condition. Additionally, all three conditions emerged with the same frequency immediately after the cue presentation.

**Figure 1:**
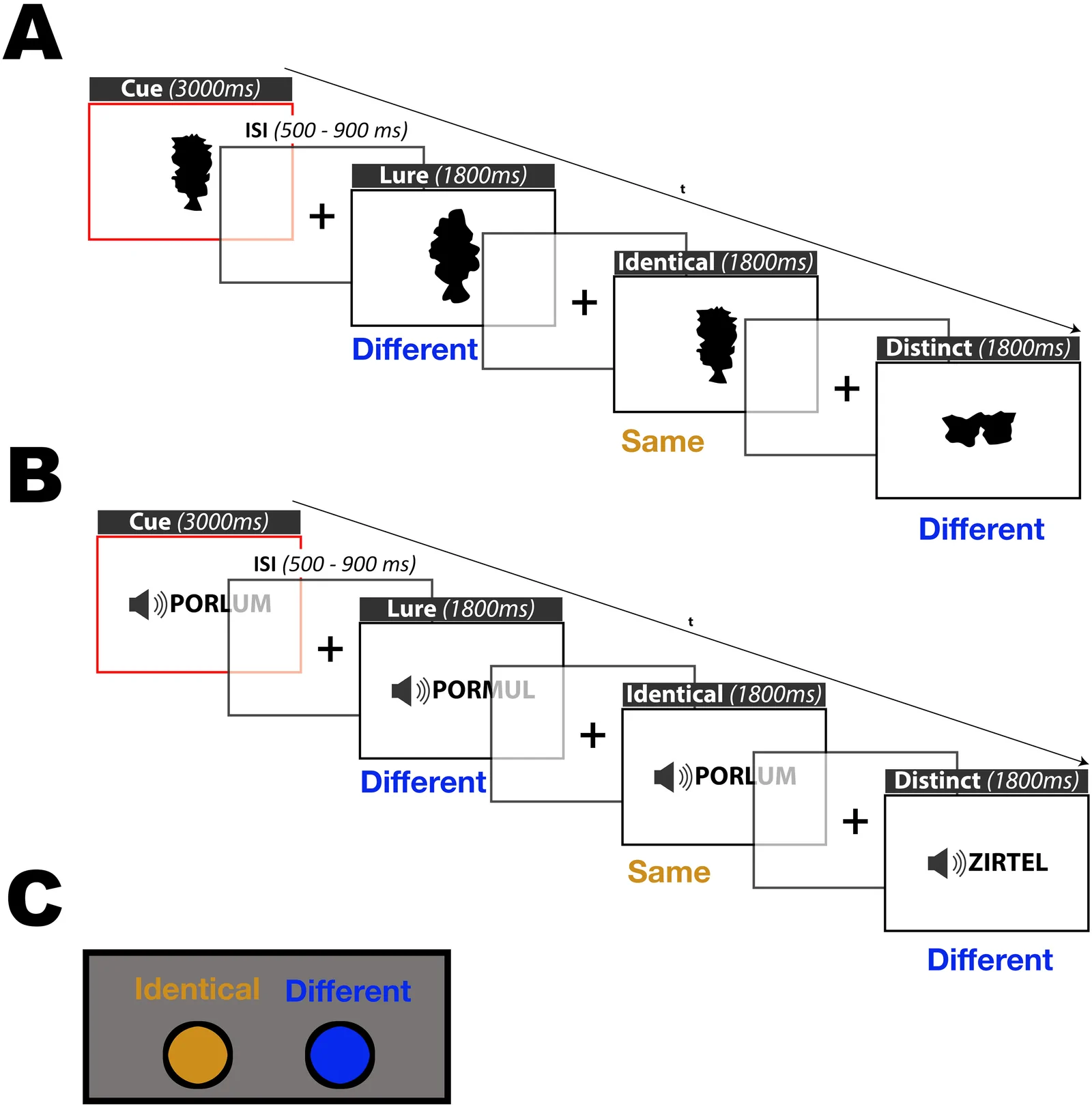
Experimental procedure for both visual **(A)** and auditory **(B)** PS. Participants were instructed to judge whether a probe stimulus was the same or different in comparison to the cue, that was presented at the beginning of each trial. Each participant’s response was recorded using a two-button panel **(C)**. To improve event separation, the inter-stimulus interval (ISI) was systematically jittered between 500ms and 900 ms. Probes were either, identical repetitions, distinct or lure images. Stimuli were presented either visually on a screen or acoustically via headphones.

### 2.4 Stimulus

For the visual task, abstract stimuli provided by Koutstaal et al. (24) were adapted by recolouring them in black and white. All pictures were in JPG format, with dimensions of 612 × 612 and displayed at a native resolution of 240 × 240. The original picture set consisted of twelve similar-looking stimuli. The perimeter of each individual stimulus was subtracted from the overall mean perimeter of the picture set. We used this method to objectively select a subsample of 7 stimuli for inclusion in the analysis. Accordingly, the stimulus with the closest-to-average perimeter served as the cue, while the other six served as probes.

For the auditory task, a set of pseudowords was selected as stimuli, as provided by Schröger et al. (25). To create lures, we transformed these pseudowords utilising the Wuggy multilingual pseudoword generator (26). The orthographic Levenshtein distance was used to objectively determine the six most distinct lures. This was achieved by measuring the absolute difference between the distances of each generated lure to the rest of the lure stimuli. From that selection, the three most distinct lures served as cues. As in the visual stimulus selection, the most distinct stimuli among the three sets were selected as the most similar-looking within each set. Each auditory stimulus was spoken and recorded by a trained speaker in WAV format (16-bit depth, 48.0 kHz sample rate).

To conclude the process of generating visual and auditory stimuli, three distinct cues were objectively selected, each accompanied by six corresponding probes for use in the experiment across both modalities.

### 2.5 Image acquisition and preprocessing

Imaging was performed at 3T (Magnetom Prisma Siemens Healthcare, Erlangen, Germany) using a 20-channel receive head coil.

T1-weighted structural data were obtained using a three-dimensional magnetisation-prepared rapid gradient-echo (MPRAGE) pulse sequence (27). Images had an isotropic resolution of 1 mm (176 in the sagittal plane, repetition time (TR) = 2000ms; echo time (TE) = 3.37 ms; flip angle (FA) = 8°; base resolution = 256 × 176 mm).

Functional T2*-weighted image acquisition was conducted via blood-oxygenation-level-dependent (BOLD) sensitive, interleaved gradient echo-planar imaging (EPI). Images had a spatial resolution of 3 × 3 mm^2^ with a slice thickness of 3.0 mm (39 transversal slices, TR = 2500 ms; TE = 30 ms; FA = 82°; base resolution = 228 × 228 mm^2^).

Image preprocessing and subsequent fMRI data analysis were performed using SPM12 (https://www.fil.ion.ucl.ac.uk/spm), which was run on MATLAB R2016b (MathWorks, Natick, MA, USA). All volumes were field map distortion corrected, slice time corrected, realigned to the first volume, coregistered to the anatomical volume, normalised to the MNI305 T1 template, and finally smoothed with a 6mm (FWHM) isotropic Gaussian kernel.

### 2.6 Behavioural analysis

To assess differences in task-related behaviour, a behavioural pattern separation score (BPS), as described in Stark et al. (3), was calculated by subtracting the sum of incorrect Lure trials (old response to lures) from the sum of correctly rejected lures (new response to Lure, correct responses) (Lure_correct responses_ – Lure_false alarms_). In addition to the BPS score, we calculated the discriminability index d’ to better control for new response biases toward similar lures and same-item probes, paralleling the contrast used during the subsequent fMRI analyses (3). For both performance estimates, a 2 × 2 repeated measures ANOVA was performed with Group and Modality as factors with two levels each (factor 1: old, young; factor 2: visual, auditory). Subsequently, *post hoc* comparisons were conducted using Tukey’s HSD to assess mean differences between factor levels.

Furthermore, Stark et al. (3) suggest calculating corrected recognition memory (REC) performance, which contrasts with the BPS metric. More specifically, previous investigations of ageing demonstrated that while BPS performance was significantly affected by age, REC remained largely unaffected. REC can be computed by subtracting the ratio of incorrect Foil trials (old response to Foils) from the ratio of correct Target trials (old response to Target). Analogous to the BPS analysis, a 2 × 2 repeated measures ANOVA was performed with Group and Modality as factors with two levels each. Mean differences were assessed using Tukey *post hoc* comparisons. The behavioural analysis, including the results visualisation, has been done using RStudio (Posit team (2025). RStudio: Integrated Development Environment for R. Posit Software, PBC, Boston, MA. URL http://www.posit.co/).

### 2.7 fMRI analysis

#### 2.7.1 Subject-level analysis

First-level analyses were conducted to assess subject and modality-specific PS effects. The individual onset times for each of the six task conditions, including their respective task durations, were convolved with a canonical hemodynamic response function (HRF). As for participants’ responses, only correct responses to all three conditions have been entered as regressors of interest. False response trials have been modelled as well but treated as a nuisance regressor. This was done to accurately separate PS-related neural activity from potential noise. The general linear model further included regressors for text display, cue, and fixation crosses, along with their respective durations. Button presses were modelled using stick functions. A first-order autoregressive model was applied to remove serial correlation, and a 128 s high-pass filter was used to remove low-frequency noise. In Addition, three movement and three rotation parameters were considered nuisance covariates and included to account for head motion. To ensure comparable activation with previous evidence (3, 4, 7), the following contrast estimates were calculated for each subject and included in the subsequent group analysis: (Lure_visual_ – Same_visual_; Lure_auditory_ – Same_auditory_).

#### 2.7.2 Group-level analysis

The second-level analyses consisted of one-sample t-tests, which were applied to test modality-specific neural activation for auditory and visual PS separately and in a direct, modality-wise comparison. For this first analysis, we considered all subjects to explore neural signals across both sensory modalities and to test for commonalities and differences, before examining age-specific effects.

To assess age effects, we entered the subject-specific contrast images for young and old subjects separately to investigate differences between visual and auditory PS using a one-sample t-test. Lastly, to inferentially confirm the effects of ageing identified in the previous step, the two groups were compared using two-sample t-tests and assessed during both visual and auditory PS.

For visual PS, we specifically hypothesised activation in the hippocampus, based on the previous literature. Therefore, to test our hypothesis, a small-volume correction (SVC) was applied using an anatomical mask generated with the SPM Anatomy Toolbox (28–30) (see **Figure 2**). Accordingly, we applied an SVC with a minimum p-value threshold of 0.05, family-wise error corrected (FWE) at the peak-voxel level, including a cluster-forming threshold *k_E_* of 30 voxels. For a step-by-step description of the present approach, we refer to the following instructions found in the SPM Mailing list (https://www.jiscmail.ac.uk/cgi-bin/webadmin?A2=spm%3B614fe6e5.1504).

**Figure 2:**
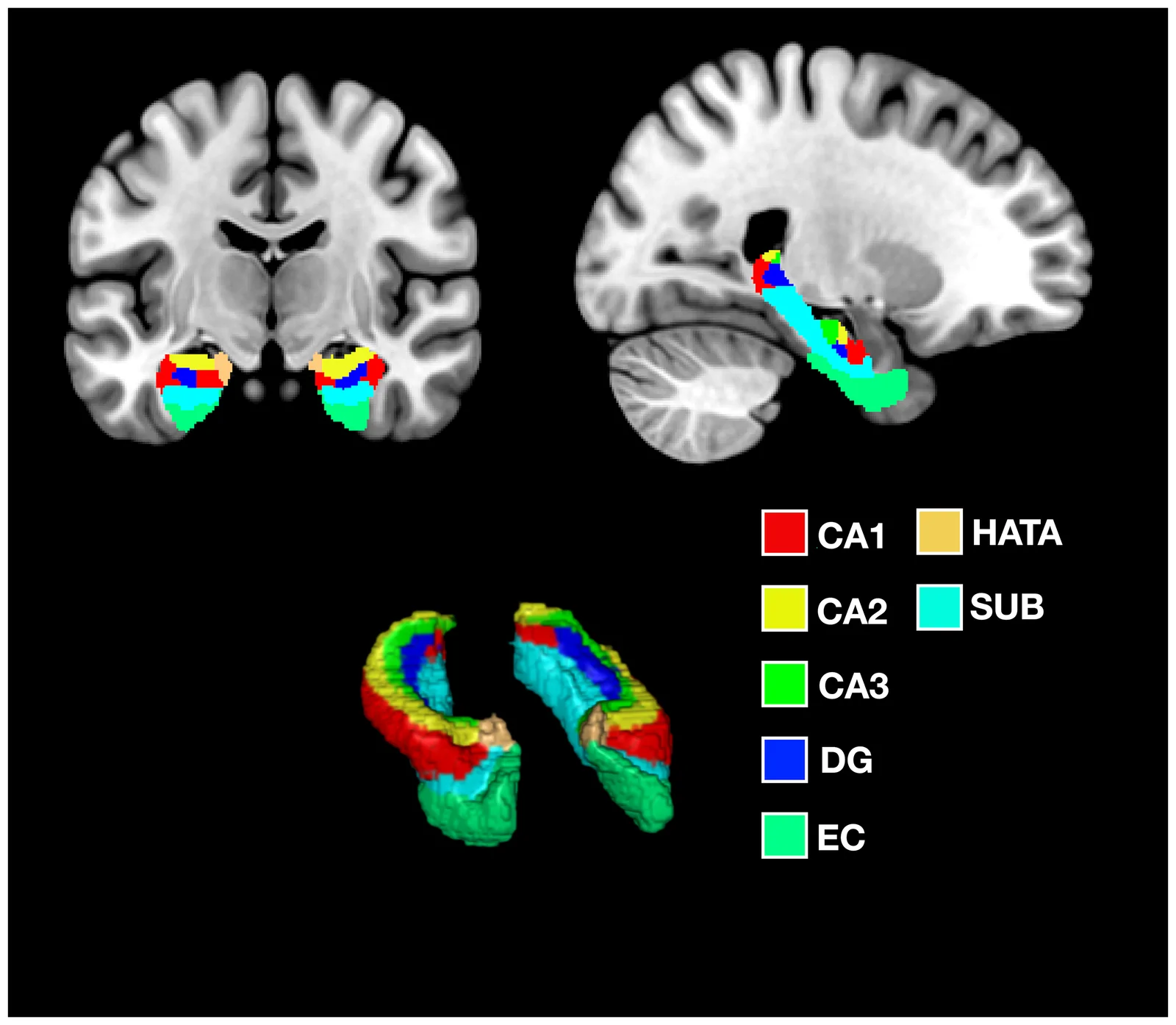
Bilateral Hippocampus mask utilised in the present study for SVC. The mask consists of regions CA1, 2 and 3, dentate gyrus (DG), entorhinal cortex (EC), hypocampo-amygdalar transition area (HATA) and subiculum (Sub). The mask was built and extracted as a single mask image using the cytoarchitectonic brain atlas provided by the Julich Brain Anatomy Toolbox for SPM12.

For exploratory reasons, and since no hypothesis for the auditory modality can be conclusively formulated, whole-brain (WB) activations for both visual and auditory PS are reported using an FWE-corrected p-value threshold of 0.05 and a minimum cluster size of 10 voxels. The final results have been visualised using MRIcroGL (31).

## 3 Results

### 3.1 Behavioural results

#### 3.1.1 BPS performance

The repeated-measures ANOVA revealed significant main effects for both group (F_(45,1)_ = 16.701, p < 0.001, η²_p_ = 0.27 [0.10, 1.0]) and modality (F_(45,1)_ = 30.889, p < 0.001, η²_p_ = 0.41 [0.23, 1.0]). The interaction effect of Group × Modality did not reach significance (F_(45,1)_ = 1.587, p = 0.246, η²_p_ = 0.03 [0.00, 1.0]).

*Post hoc* comparisons using least squares means revealed significant within- and between-group differences. The elderly group demonstrated significantly better performance during visual compared to auditory PS (mean_visual_ = 0.782, SD_visual_ = 0.037; mean_auditory_ = 0.560, SD_auditory_ = 0.051; *p* < 0.001, *t* = 4.873). The young group performed significantly better during visual than auditory PS (mean_visual_ = 0.961, SD_visual_ = 0.037; mean_auditory_ = 0.821, SD_auditory_ = 0.052; *p* = 0.022, *t* = 3.007). Between the two groups, the young significantly outperformed the elderly group both during visual (mean_young_ = 0.961, SD_young_ = 0.037; mean_old_ = 0.782, SD_old_ = 0.037; *p* = 0.007, *t* = 3.442) and auditory PS (mean_young_ = 0.821, SD_young_ = 0.052; mean_old_ = 0.560, SD_old_ = 0.051; *p* = 0.004, *t* = 3.641) (see **Figure 3**).

**Figure 3:**
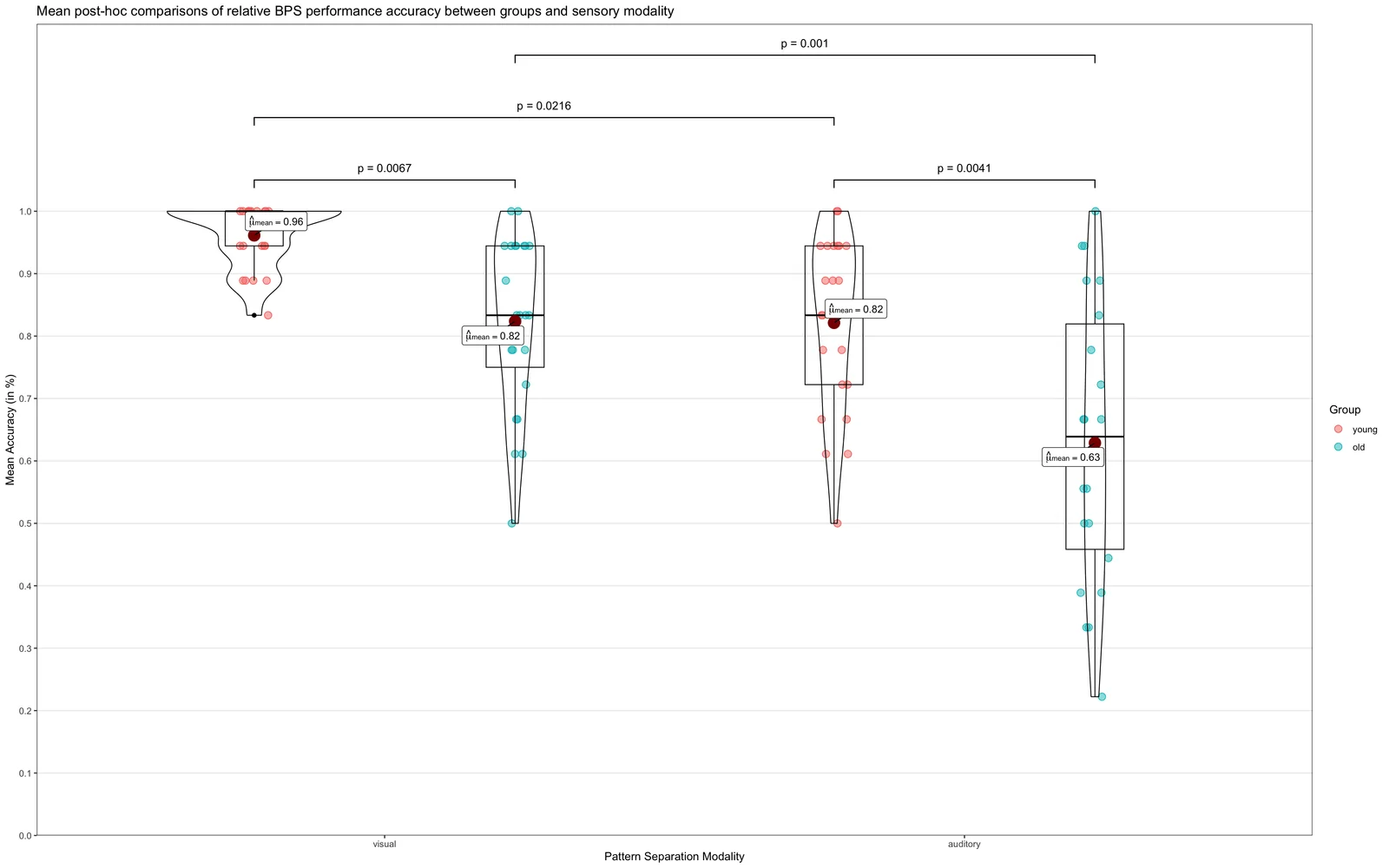
Combined plot showing *post-hoc* comparisons of BPS performance accuracy (in percentage) using Tukey’s HSD to test for significant differences due to group and modality.

#### 3.1.2 Bias-corrected discriminability performance d’

The repeated-measures ANOVA revealed significant main effects for both group (*F*_(45,1)_ = 21.751, *p* < 0.001, *η²_p_* = 0.32 [0.14, 1.0]) and modality (F_(45,1)_ = 12.461, p < 0.001, *η²_p_* = 0.2 [0.05, 1.0]). The interaction effect of Group × Modality did not reach significance (*F*_(45,1)_ = 1.354, *p* = 0.251, *η²_p_* = 0.02 [0.00, 1.0]).

*Post hoc* comparisons revealed that the young did not differ significantly between visual and auditory PS (mean_visual_ = 0.929, SD_visual_ = 0.028; mean_auditory_ = 0.877, SD_auditory_ = 0.028, *p* = 0.359, *t* = 1.656), whereas the old group performed significantly worse during auditory than visual PS (mean_visual_ = 0.792, SD_visual_ = 0.031; mean_auditory_ = 0.689, SD_auditory_ = 0.03; *p* = 0.009, *t* = 3.355). The young group still significantly outperformed the old group during visual (mean_young_ = 0.929, SD_young_ = 0.028; mean_old_ = 0.792, SD_old_ = 0.031; *p* = 0.006, *t* = 3.482) and auditory PS (mean_young_ = 0.877, SD_young_ = 0.028; mean_old_ = 0.689; SD_old_ = 0.03; *p* > 0.001, *t* = 4.381), respectively (see **Figure 4**).

**Figure 4:**
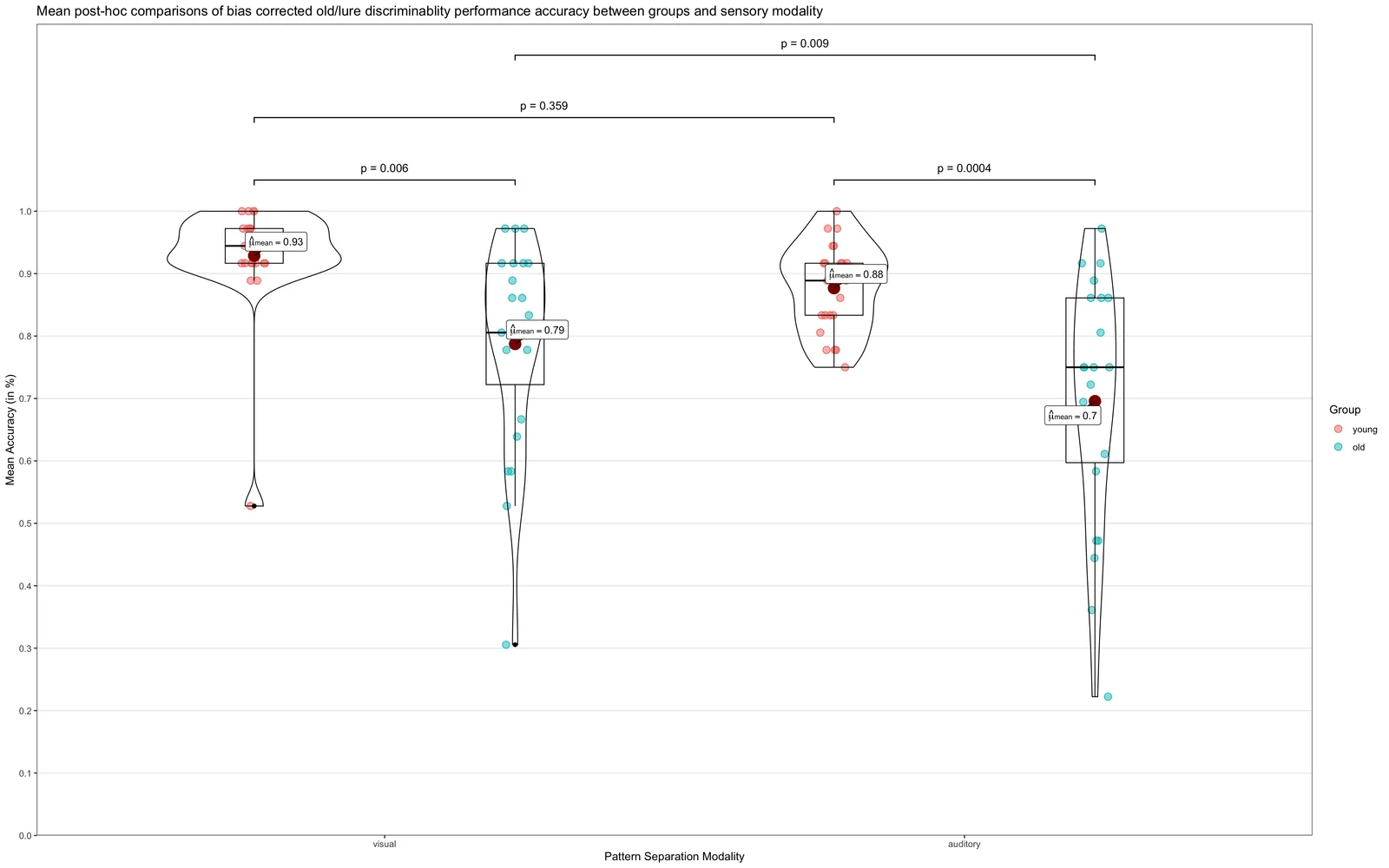
Combined plot showing *post-hoc* comparisons of bias-corrected old/lure discriminability (d’) performance accuracy (in percentage) using Tukey’s HSD to test for significant differences due to group and modality.

#### 3.1.3 REC performance

The REC performance differed significantly between the two groups (F_(45,1)_ = 19.201, p < 0.001, η²_p_ = 0.30 [0.13, 1.0]). The main effect of modality (F_(45,1)_ = 0.012, p = 0.914, η²_p_ = 0.0003 [0.00, 1.0]) and the interaction effect of Group × Modality (F_(45,1)_ = 0.595, p = 0.445, η²_p_ = 0.01 [0.00, 1.0]) did not reach significance.

*Post hoc* comparisons using least squares means revealed significant between-group but not within-group differences. The young group significantly outperformed the elderly group during both visual (mean_young_ = 0.937, SD_young_ = 0.034; mean_old_ = 0.772, SD_old_ = 0.034; *p* = 0.006, *t* = 3.455) and auditory PS (mean_young_ = 0.954, SD_young_ = 0.031; mean_old_ = 0.759, SD_old_ = 0.030; *p* < 0.001, *t* = 4.560). However, the young (mean_visual_ = 0. 0.937, SD_visual_ = 0.034; mean_auditory_ = 0.954, SD_auditory_ = 0.031; *p* < 0.927, *t* = 0.616) and elderly group (mean_visual_ = 0.772, SD_visual_ = 0.034; mean_auditory_ = 0.759, SD_auditory_ = 0.030; *p* < 0.965, *t* = 0.474) did not show significant performance differences between visual and auditory PS (see **Figure 5**).

**Figure 5:**
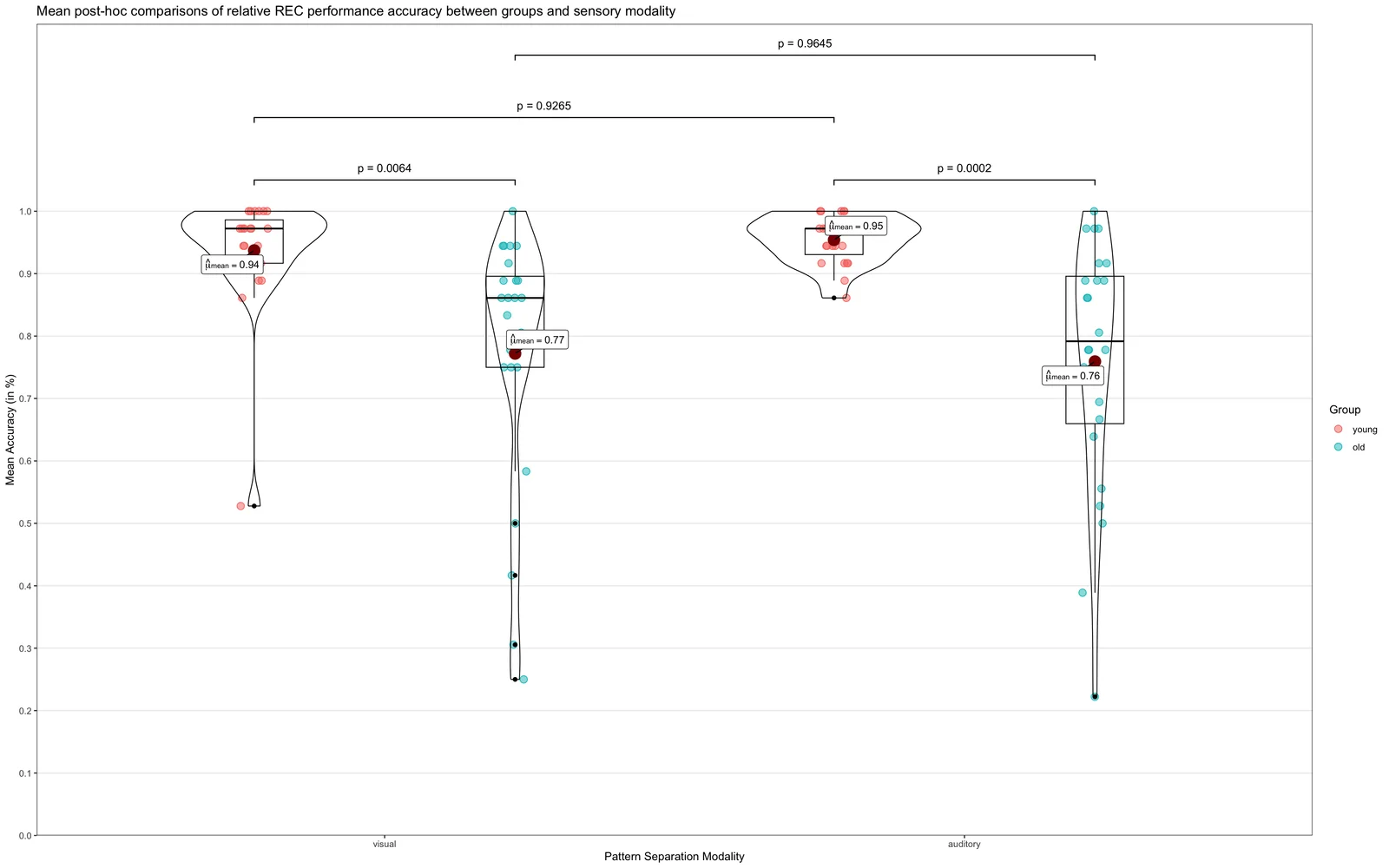
Combined plot showing *post-hoc* comparisons of REC performance accuracy (in percentage) using Tukey’s HSD to test for significant differences due to group and modality.

### 3.2 Imaging results

#### 3.2.1 Modality comparison - whole group analysis

The visual PS contrast revealed two significant bilateral posterior Hippocampus clusters under SVC_FWE_ = 0.05. No suprathreshold activation was found outside of the Hippocampus under whole-brain FWE correction = 0.05 (see **Table 1**).

**Table 1:** Neural activation of visual pattern separation in the whole sample under SVC, using the bilateral hippocampus mask, and WB.

						MNI Coordinates			
Contrast	Clusterdefining -threshold	Region	Hem.	k	T	x	y	z	p-value
Lure > Same	SVC at 4.439; k_E_ = 30	**Hippocampus (DG)**	R	36	5.88	28	-34	-8	**0.001**
		**Hippocampus (DG)**	L	39	5.50	-22	-38	6	**0.003**
	WB at 5.70; k_E_ = 10	No suprathreshold clusters present during whole-brain correction							

SVC, small volume correction; WB, whole-brain correction. Significant clusters are highlighted in bold; not significant clusters are labelled as n.s.

In Contrast, during auditory PS no activation was found under bilateral Hippocampus SVC_(FWE = 0.05)_. WB_(FWE = 0.05)_ correction revealed activation of two clusters in the superior temporal gyrus, including activation clusters in the pre-supplementary motor area, precentral gyrus and the inferior frontal gyrus, pars Opercularis (see **Table 2**).

**Table 2:** Neural activation of auditory pattern separation in the whole sample under small-volume correction for the bilateral hippocampus and whole-brain correction.

						MNI Coordinates			
Contrast	Clusterdefining -threshold	Region	Hem.	k	T	x	y	z	p-value
Lure > Same	SVC at 5.158; k_E_ = 30	No suprathreshold clusters present during small volume correction							
	WB at 5.750; k_E_ = 10	**Superior temporal gyrus**	L	416	8.58	-56	-36	2	**<0.001**
		**Pre supplementary motor area (Pre- SMA)**	L	44	6.74	-6	14	52	**0.003**
		**Precentral gyrus**	L	10	6.31	-48	-2	48	**0.010**
		**Superior temporal gyrus**	L	12	6.18	-54	-16	-6	**0.014**
		**Inferior frontal gyrus, pars opercularis**	L	12	6.13	-50	18	26	**0.017**

SVC, small volume correction; WB, whole brain. Significant clusters are highlighted in bold not significant clusters are labelled as n.s.

#### 3.2.2 Group-wise modality comparisons

##### 3.2.2.1 One-sample t-test per group and PS modality

During visual PS, the young group did not show significant activation in the Hippocampus following SVC. However, we did find significant bilateral posterior hippocampal activation in the elderly group following SVC (see **Table 3**).

**Table 3:** Neural activation of visual pattern separation was separated for the young and elderly groups under small-volume correction for the bilateral hippocampus and whole-brain correction.

							MNI Coordinates			
Group	Contrast	*Clusterdefining -threshold*	Region	Hem.	k	T	x	y	z	*p-value*
Young	Lure > Same	SVC at 5.077; kE = 30	Hippocampus	L	36	4.86	-26	-40	-4	0.076 (n.s.)
			Parahippocampal Gyrus	L	120	4.70	-18	-18	-22	0.100 (n.s.)
		WB at 7.016; kE = 10	No suprathreshold clusters present during whole-brain correction							
Elderly	Lure > Same	SVC at 5.111; kE = 30	**Hippocampus**	L	23	6.70	-24	-36	0	**0.002**
			**Hippocampus**	R	22	6.09	30	-33	-8	**0.008**
			Hippocampus	L	31	4.10	-27	-33	-10	0.297 (n.s.)
			Hippocampus	R	33	4.10	22	-15	-24	0.300 (n.s.)
		WB at 7.085; kE = 10	**Lateral occipital cortex, inferior division**	R	17	8.85	45	-60	-2	**0.002**

SVC, small volume correction; WB, whole brain. Significant clusters are highlighted in bold not significant clusters are labelled as n.s.

During auditory PS, neither group showed significant hippocampal activation following SVC. Under FEW-correction, the whole-brain analysis revealed significant activation in the left Superior temporal gyrus and left inferior frontal gyrus, pars Opercularis, in the young group. In the elderly group, we found significant activation of the left superior temporal gyrus. The cluster, however, lies in the posterior division, unlike that in the young group, which is situated in the anterior division of the superior temporal gyrus (see **Table 4**).

**Table 4:** Neural activation of auditory pattern separation was separated for the young and elderly groups under small-volume correction for the Bilateral Hippocampus and whole-brain correction.

							MNI Coordinates			
Group	Contrast	*Clusterdefining -threshold*	Region	Hem.	k	T	x	y	z	*p-value*
Young	Lure > Same	SVC at 5.158; kE = 30	No suprathreshold clusters present during small volume correction							
		WB at 7.120; kE = 10	**Inferior frontal gyrus, pars opercularis**	L	48	9.50	-48	15	28	**0.001**
			**Superior temporal gyrus**	L	24	8.39	-56	-27	-4	**0.001**
Elderly	Lure > Same	SVC at 5.111; kE = 30	No suprathreshold clusters present during small volume correction							
		WB at 7.085; kE = 10	**Superior temporal gyrus**	L	19	8.20	-57	-39	3	**0.008**

SVC, small volume correction; WB, whole brain. Significant clusters are highlighted in bold not significant clusters are labelled as n.s.

##### 3.2.2.2 Direct group comparisons

During visual PS, no significant activation was found in either the SVC or the subsequent whole-brain analysis for either group contrast (see **Table 5**). Likewise, during auditory PS no activation was found for both group contrasts under SVC or whole-brain correction (see **Table 5**).

**Table 5:** Neural activation of visual pattern separation in a direct group comparison, both under small-volume correction for the bilateral hippocampus and whole-brain correction.

							MNI Coordinates			
Group comparison	Contrast	*Clusterdefining -threshold*	Region	Hem.	k	T	x	y	z	*p-value*
Young > Elderly	Lure > Same	SVC at 4.453; kE = 30	No suprathreshold clusters present during small-volume correction							
		WB at 5.726; kE = 10	No suprathreshold clusters present during whole-brain correction							
Elderly > Young	Lure > Same	SVC at 4.453; kE = 30	Hippocampus	R	45	3.98	27	-33	-3	0.162 (n.s.)
		WB at 5.726; kE = 10	No suprathreshold clusters present during whole-brain correction							

SVC, small volume correction; WB, whole brain. Significant clusters are highlighted in bold not significant clusters are labelled as n.s.

## 4 Discussion

In this study, we investigated the neural underpinnings of auditory PS for the first time in direct comparison with those of visual PS, using an adaptation of the MST task developed by Stark et al. (3). Firstly, the present paradigm successfully replicated hippocampal involvement, i.e., DG activity, during visual PS, as demonstrated numerous times in previous studies on PS. During our initial whole-group analysis, we found two bilateral dorsal hippocampal clusters. However, this effect was not consistent across age groups; only the older group showed bilateral Hippocampal activation compared to the younger group. Secondly, and most strikingly, no hippocampal activation was observed during auditory PS, in both our whole-group analysis and our age analyses. Instead, the auditory PS contrast revealed multiple left-hemispheric clusters in the superior temporal gyri, pre-SMA, and inferior frontal gyrus, pars opercularis. Beyond the hippocampus, whole-brain analyses demonstrated lateral occipital activation only in the old group during visual PS. In sum, the direct comparison of modalities across the visual and auditory domains demonstrates distinct, possibly modality-specific recruitment within the two neural PS systems. These findings are discussed further below.

### 4.1 No hippocampus during auditory PS

The demonstrated differing activation patterns between visual and auditory PS raises the question of whether the hippocampus plays any role in discriminating highly similar auditory stimuli. As outlined in the introduction, most studies on PS have used the Mnemonic Similarity Task by Stark et al. (3, 32), with many adaptations made over the years. However, it remains unclear which specific characteristics of MST paradigms lead to hippocampal activation, or a lack thereof, and are thus associated with successful PS performance across different sensory domains.

A possible explanation could lie in the specific neural mechanisms underlying the processing of non-semantic visual and auditory stimuli. The hippocampus’s role in visual perception and the discrimination of visual imagery has since been well established (33). The majority of evidence on PS has been acquired in the context of visual perception of everyday objects, scenes or faces (14, 34, 35). In a compelling review, Liu et al. (36) suggest that abstract images may be more favourable than familiar everyday objects for detecting and validating PS. The reason is that PS paradigms that use familiar everyday objects cannot fully rule out the influence of other memory-related processes, such as familiarity-driven recognition and gist perception, which have been shown to engage different regions of the hippocampus (37–39). Interestingly, a recent fMRI study used non-semantic images to assess object and spatial PS in the visual domain. The perirhinal cortex was significantly activated during object recognition, while the parahippocampal gyrus was significantly activated during spatial PS. Interestingly, the CA3 and DG did not differ significantly in BOLD signal change when separately assessing Lures and Repetitions, whereas the perirhinal cortex did (40). The present findings contrast with the aforementioned findings and facilitate DG involvement during PS of abstract images (see **Figure 6**). However, further studies are needed to determine whether the CA3/DG serves as an exclusive and universal pattern separator.

**Figure 6:**
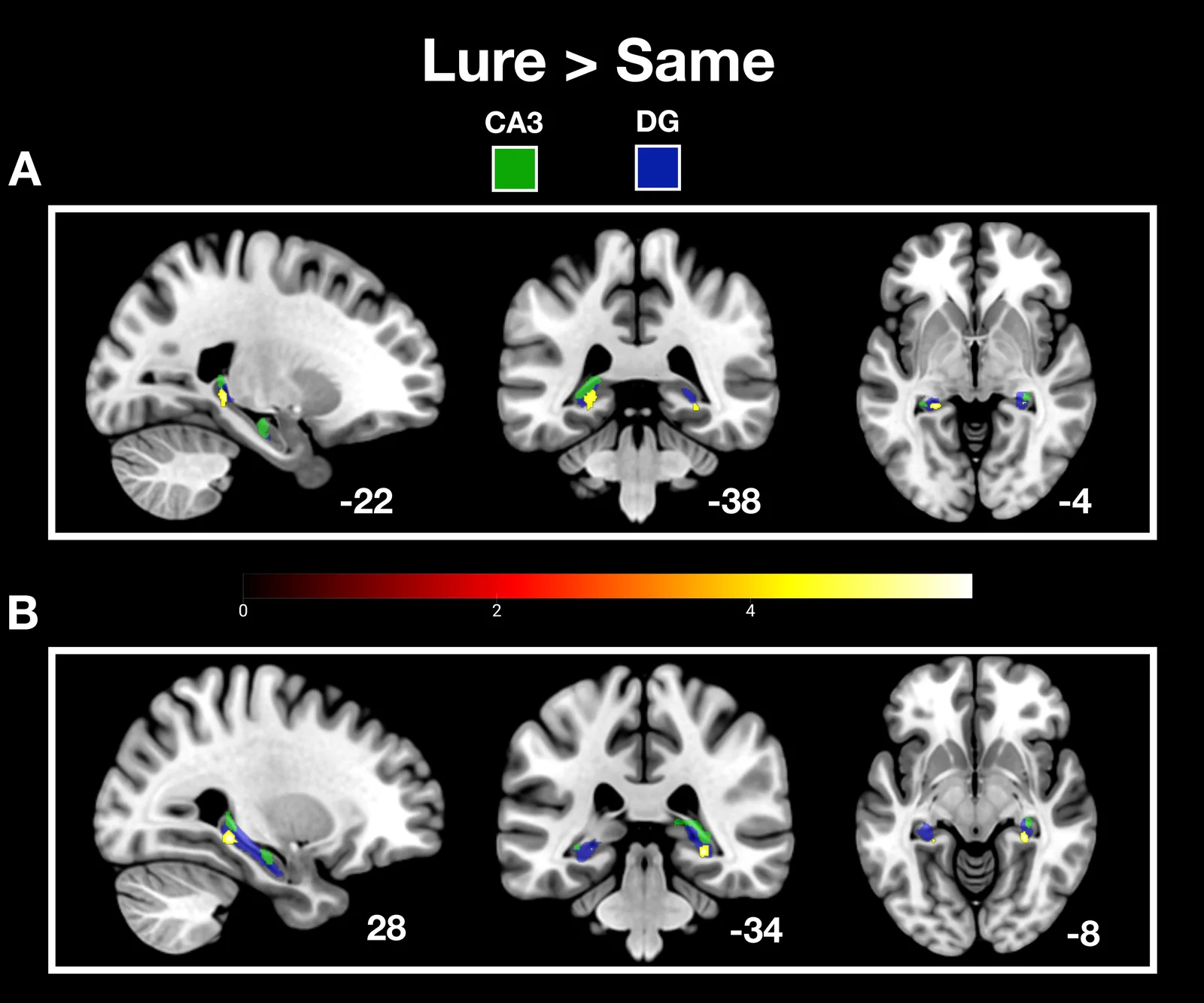
Left **(A)** and right **(B)** DG activation observed during visual PS under the contrast: Lure > Same (SVC pFWE = 0.05). Bilateral DG (blue) and CA3 (green) masks were included to highlight hippocampus activations. The images are displayed in left and right sagittal, axial and coronal planes in that order. The activation strength is depicted through the centred activation bar.

Compared to the visual system, growing evidence has implicated hippocampal function in auditory perception of sounds, words, and pseudowords as well (cf. 41). In accordance, a recent case study by Wang et al. (42) involving subject B.L., who suffered highly selective bilateral lesions of the DG, tested auditory PS performance behaviourally using a trisyllabic pseudoword and sound MST paradigm, and compared the effects with those of a healthy control group. The results demonstrated substantial performance impairments in both the pseudoword and the sound discrimination tasks. In particular, B.L. exhibited a response bias towards “old” judgments to lures, similar to the performance bias found in older subjects (43). Thus, the authors concluded that structural impairments to the DG correlate with impairments during behavioural auditory PS. Furthermore, previous studies on pseudoword encoding and discrimination further corroborated the importance of the hippocampus. Although speculative, one possible explanation could be that the Hippocampus demonstrates hypoactivation when learning artificial rules, i.e., grammar and words, whereas the IFG tends to exhibit hyperactivation (44). This activation, an Interaction between the IFG and the hippocampus, is attributed to similarity-based learning. Hippocampal hyperactivation apparently determines the initial state during learning of artificial grammar or words and decreases over time. In other words, once the familiarisation with the new rules and words has been achieved, the hippocampus appears to shift from a state of hyper- to hypoactivation over time (44). This could, in part, explain why no hippocampal involvement was observed during auditory PS in the present study. Recent accounts of pseudoword processing confirm that the hippocampus shows decreased relative activation strength with continued learning and familiarisation of the pseudowords (45). This finding appears consistent when comparing novel pseudowords with previously learned ones or with those paired with rich, meaningful objects (46). Therefore, the hippocampus may deactivate during learning of new non-semantic words, compared with meaningful words or episodically relevant auditory stimuli.

### 4.2 Auditory PS might be functionally related to linguistic (pseudo)word processing, lexical ambiguity resolution and voluntary motor control

During visual PS, we observed significant bilateral posterior/dorsal hippocampal activation.

Contrary to our previous hypothesis, no hippocampal activation was present during auditory PS. Instead, multiple left-hemispheric cortical regions were identified during auditory PS, namely the middle and posterior superior temporal gyrus (STG), the pre-supplementary motor area (pre-SMA) and the inferior frontal gyrus (IFG) (see **Figure 7**). Traditionally, the STG has been linked to the extraction of meaningful linguistic information from auditory input and shows differential engagement along the anterior-posterior axis (47, 48). Specifically, the middle part of the STG appears to process syllable-level coding and continuous speech input, while the posterior STG engages with speech onset (48). In addition, the left posterior STG, in particular, has been shown to engage after repeated exposure to pseudowords (49). The STG in particular appears to be part of the anteroventral auditory processing stream. In its role, it decodes the meaning of a particular sound before relaying that information to the IFG (50).

**Figure 7:**
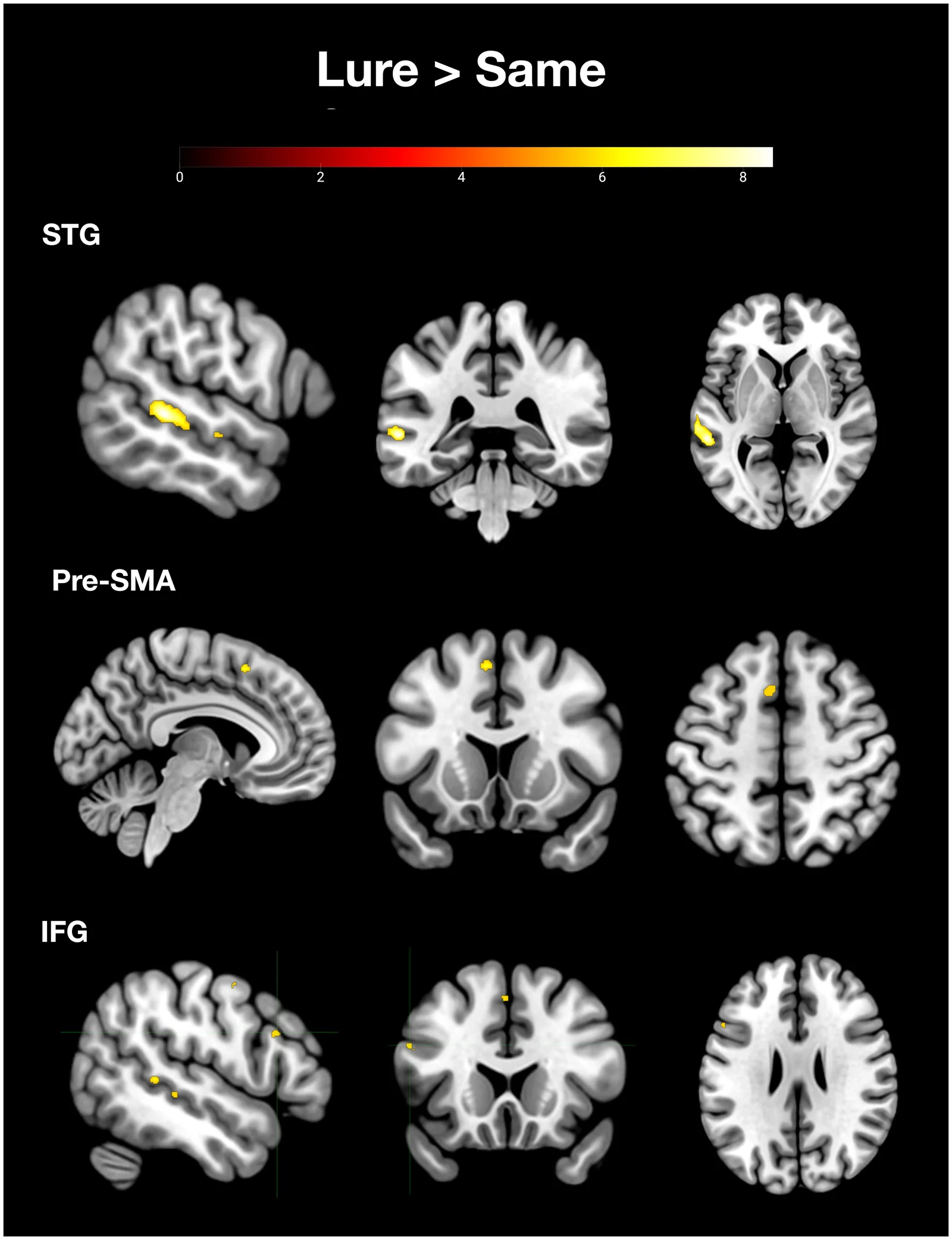
Significant left hemispheric cortical activation in the STG, pre-SMA and IFG found during auditory PS under the contrast: lure > same (WB pFWE = 0.05). The images are displayed in left sagittal, coronal and axial planes. To aid cluster identification, a green crosshair has been added to the sagittal and coronal images of the IFG. The activation strength is depicted through the top activation bar.

The IFG, a region corresponding to Broca’s area (BA44/45), can be attributed to speech and overall language processing (51), and it appears that increased left IFG activation, in particular, is associated with resolving interference between phonetically similar words (52) and pseudowords (53). This region appears to be functionally and structurally related to the pre-SMA. Accordingly, the pre-SMA shows strong structural and functional connectivity with other language centres in the brain, particularly the IFG and STG (50). Both the pre-SMA and the IFG are directly connected via the frontal aslant tract, a connection that is important for supporting language and speech-related functions, as well as executive functions such as inhibitory control and conflict resolution of voluntary actions (54). Accordingly, the pre-SMA has traditionally been linked to motor response inhibition and serves as a central region for the executive guidance of attention during the processing of words or pseudowords (55). In recent years, it has also been linked to the sequential encoding of auditory stimuli and integrating these linguistic stimuli to form more complex auditory representations, i.e., from syllables to words and full sentences (56). The pre-SMA appears to be activated regardless of the modality of comprehension (visual or auditory) or production (spoken or written) (56). In relevance to our investigation, pre-SMA activity has been specifically linked to the discrimination of overlapping auditory representations, including task initiation and switching (57). Accordingly, improving motor response inhibition in patients with obsessive-compulsive syndrome has been shown to increase structural connectivity between the IFG and pre-SMA (58). Therefore, it is possible that the pre-SMA and the IFG may be equally involved not only in processing auditory stimuli but also in resolving interference from overlapping auditory representations, while receiving information about auditory information from the STG. In addition, all three regions have been observed exclusively in the left hemisphere. This finding is consistent with the lateralisation hypothesis in speech-related processing, in which humans tend to show activation in speech- and auditory-processing regions contralateral to their dominant hand (59, 60). Taken together, it is possible that the present findings reflect a functional overlap between purely auditory PS and language-specific neural mechanisms, such as general auditory (pseudo)word encoding, resolving lexical interference of similar-sounding stimuli, and voluntary motor response inhibition to auditory stimuli, which occur predominantly in the left hemisphere.

### 4.3 BPS and REC are impaired in older subjects

On a behavioural level, the older subjects’ performance on BPS and REC was significantly impaired compared with that of the young subjects. This age effect remained, even after correcting for a potential response bias. However, the present findings, in part, contrast with previous studies on age-related visual PS performance, which did not observe a decline in REC performance in elderly subjects. However, a more recent study by Wang et al. (61) investigated age-related behavioural PS performance deficits in young and older subjects using a trisyllabic pseudoword auditory MST task. The authors found that the older group performed significantly worse during MST, on both lure discrimination and recognition memory. In addition, the authors did not find differences in the statistical learning task, in which the same participants were exposed to a series of new pseudowords, followed by an implicit recognition task, with either the same word, a partword, consisting of two previously heard words, or a completely new word. Conversely, the older subjects were as good as the younger subjects at differentiating between the same words heard, part-words, and new words. However, when explicitly asked to rate the familiarity of a particular stimulus, age effects reappeared, similar to those observed in the MST task (23).

These findings are consistent with previous accounts on implicit (i.e., incidental) and explicit memory performance effects found in elderly subjects (62–65). While explicit memory conditions involve conscious recollection and recognition, implicit memory conditions are, by nature, unconscious and procedural and can be evoked through priming (66). Performance of elderly subjects under implicit conditions, i.e., reduced mnemonic or instructional load, mitigates age-related performance issues in memory tasks (67, 68). Accordingly, performance in older subjects decreases when they are explicitly asked to perform a task rather than simply to repeat an instruction or to recall it verbally (69). In the context of PS, implications for future practice encourage adapting future MST versions to reduce mnemonic load, particularly in the context of aging, to further delineate effects based on pattern separation, e.g., through priming (17, 36).

### 4.4 Limitations

The present study has several limitations and challenges, particularly in imaging older subjects. Ageing is associated with increased interindividual variability in cognitive and physical abilities, vascular health, and neural integrity, all of which may reduce the sensitivity of age comparisons in fMRI studies (70, 71). In addition, age-related sensory decline in the visual and auditory domains may introduce variance unrelated to mnemonic PS (72–74). Decline in one perceptual domain often extends to the other with advancing age (75). Moreover, both the primary visual cortex and the left temporal gyrus show reduced BOLD signal activation in healthy older adults with otherwise sufficient perceptual abilities (73, 76). Older subjects are also more susceptible to head movement and altered neurovascular coupling, both of which can introduce noise into BOLD data (77, 78). Although no participant in the present study exceeded 2 mm or 2 degrees of head motion, altered neurovascular coupling cannot be entirely ruled out and may have contributed to apparent hypoactivation due to reduced blood flow if not explicitly controlled for (79).

Another possibility is that the current paradigm, designed primarily to investigate visual and auditory PS differences, may not have been sufficiently sensitive to detect age-related neural changes in PS. The sample size of 46 subjects (23 per group) is comparable to previous PS studies (14, 15, 80). Stark et al. (80) examined the stability of age-related deficits across different MST adaptations and found that a gist-based continuous recognition approach improved PS performance in healthy older adults compared to a test/study design, although deficits remained. Neither reducing stimulus set size nor using a simplified two-choice (“new” vs. “old”) design eliminated age-related impairments. However, a two-choice design cannot fully delineate between actual correctly classified lures and gist-based decisions. A confidence rating could have provided further information on a subject’s decision-making during PS. Furthermore, the addition of subjective difficulty ratings could have provided further insight into the diminished REC performance in older adults, which could have been caused by increased mental load in spite of previous exposure or practice. Consistent with these findings, the present study demonstrated significant age-related deficits in behavioural PS performance. However, it remains unclear how these methodological adaptations differentially engage the neural substrates of PS across younger and older adults and across sensory domains (17, 36).

## 5 Conclusion

To conclude, the present findings point to a differential recruitment of neural substrates between visual and auditory PS. In general, the present findings support the notion that auditory PS may be functionally related to cortical regions observed during auditory pseudoword processing and, more broadly, auditory discrimination. Contrary to our expectations, we did not find prominent hippocampal BOLD signal activation during auditory PS. While the present findings demonstrate robust behavioural decline in PS and REC performance, the age-related neural changes remain inconsistent and may be attributable to confounding neurofunctional changes in neurovascular coupling and to a rapid decline in perceptual abilities, frequently observed in healthy older adults. Thus, these findings should be further investigated and, ideally, replicated to explore the age-related neural mechanisms underlying visual and auditory PS in more detail.

## Data Availability

The raw data supporting the conclusions of this article will be made available by the authors, without undue reservation.
