# Supplementary material for: Dentate Gyrus activation during visual but not auditory pattern separation: an investigation of perceptual modality differences in young and old individuals using fMRI
Source: Front Psychiatry. 2026 Sep 24;17:1900423. doi: 10.3389/fpsyt.2026.1900423 (PMC13647137; doi:10.3389/fpsyt.2026.1900423)
Supplement: Supplementary file 1 [file Table1.docx]

**Supplementary materials**

|  | Young (*N* = 23) | Old (*N* = 23) |
| --- | --- | --- |
| Age in years (mean, SD & range) | 23.391 (3.23); 19 - 31 | 68.580 (5.00); 59 - 79 |
| Sex | 7 Males (30.43%); 16 Females (69.57%) | 10 Males (45.45%); 12 Females (54.55%) |
| Handedness | 21 right-handed (91.31%) | 22 right-handed (95.65%) |
| Years of education (mean & SD) | 13.74 years (3.12) | 12.46 years (3.14) |

**Supplementary Table 1.** Demographic values of the young and old groups. Note: SD = standard deviation, *N* = sample size.
